# Special Issue “Ethnopharmacology in Latin America”

**DOI:** 10.3390/ph16091189

**Published:** 2023-08-22

**Authors:** Angel Josabad Alonso-Castro

**Affiliations:** Departamento de Farmacia, Universidad de Guanajuato, Noria Alta, Colonia Noria Alta Guanajuato, Guanajuato 36250, Mexico; angeljosabad@ugto.mx; Tel.: +52-473-732-0006

Latin America is a multicultural region encompassing 43 countries, with 665 million inhabitants with a mean age of 31 years old, 84% of whom live in urban areas [1]. Currently, many countries in Latin America experience political instability, precariousness in their health systems, drug trafficking, poverty, and other factors that affect quality of life. Latin America has a high (around 30%) prevalence of obesity due to the high availability of ultra-processed foods, demographic changes, and sedentarism [2]. Diabetes and heart diseases have a high prevalence in this region [3,4,5,6]. The increase in adult mortality in the last five years was due to heart diseases, metabolic diseases, COVID-19, and violence-related deaths [2,4,5,6]. The inhabitants from this region also present two or more chronic conditions with a prevalence of 12.4–25% and a mean age of 40 years old [3]. Access to health care is difficult and many people have problems paying medical bills [3,4]. Therefore, the population looks to traditional medicine to remedy their health issues [7]. This Special Issue, “Ethnopharmacology in Latin America”, enriches scientific knowledge of the medicinal plants of Latin America. The scientific studies herein investigate traditional medicine for sources of compounds for treating the chronic diseases that affect Latin America, such as wounds in diabetic patients [8], anxiety and depression [9], gynecological disorders, such as vulvovaginitis and endometriosis [10,11], and metabolic syndrome [12]. The scientific works in this Special Issue focus on providing solutions for current chronic diseases in this region.

Five of the eleven works published in this Special Issue focused on the anti-inflammatory effects of a pharmaceutical formulation (a vaginal gel) [10] and nanoemulsions containing plant extracts [8,11,13] and their active compounds [14]. All these formulations, plant extracts, and compounds increased the levels of the pro-inflammatory cytokine IL-10 and decreased the levels of pro-inflammatory mediators such as TNF-α, IL-1β, and nitric oxide. Most studies used in vivo models [9,10,12] and two studies assessed the pharmacological actions using in vitro models [11,14]. Preventing chronic inflammation is a key pharmacological avenue for avoiding the development of chronic diseases such as cancer, cardiovascular diseases, and neurodegenerative disorders [15].

In some studies [8,9,12,13], the extraction of medicinal plants was carried out through the use of solvents, like ethanol and water, used in traditional medicine, whereas other studies used organic solvents like acetone and dichloromethane [10,14]. This type of decision affects the type of compounds that can be obtained and identified. Medicinal plants were chemically standardized using gas chromatography-mass spectrometry [8,9] and liquid chromatography-mass spectrometry [8,12,13]. The reported secondary metabolites were phytosterols and flavonols [8], fatty acid [9], flavonoid glycosides [12,13], caffeic acid derivates [12], and terpenes [14]. Two active compounds obtained from medicinal plants were evaluated: piquerol, obtained from *Piqueria trinervia* [14], and myristic acid, obtained from *Ceiba aesculifolia* [9], indicating the need to perform pharmacological studies on the active compounds from medicinal plants. It is well-known that, on some occasions, the yield obtained from these compounds can be low. Chemical synthesis and biotechnological approaches are options for solving this problem [16]. However, no bio-directed assays were reported in this Special Issue.

Other pharmacological and pharmaceutical studies included the following. Bustos-Gomez et al. [9] concluded that the anxiolytic activity of myristic acid, from *Ceiba aesculifolia*, is mediated by the noradrenergic and serotonergic systems using in silico studies and in vivo assays. Ximenes et al. [12] showed that *Baccharis trimera* is an option for preventing metabolic syndrome because this plant extract reduced the dietary intake, weight gain, and adipose tissue accumulation in mice fed with a high-fat diet. The study of Alonso-Castro et al. [17] emphasizes the need to carry out social campaigns to decrease self-medication practices in children, because more than 50% of parents in Mexico self-medicate their children with allopathic and herbal medicine.

Three review articles were published [18,19,20]. Al-Imam et al. [18] provided information about dimethyltryptamine, an indole alkaloid found in several plant species and the Colorado River toad (*Incilius alvarius*). The authors described the historical uses, as well as pharmacological and toxicological uses of this alkaloid and discussed its legal aspects. Barreto Linares et al. [19] described the ethnobotany, phytochemistry, and pharmacology of *Schinopsis brasiliensis* and highlighted the need to perform pharmacological studies with the flavonoids and tannins, such as corilagin, identified in this tree species. Finally, Salazar-Gomez and Alonso-Castro [20] provided information on the wound-healing activity of medicinal plants from Latin America. This review underlined the need to evaluate the wound-healing activity of medicinal plants from Latin America, because 65% of the plants recorded in this review remain to be fully pharmacologically and chemically studied.

In summary, the works published in this Special Issue highlight the biological richness in Latin America, provide new information about the chemical composition of plant extracts, and give pharmacological and toxicological information on these plant extracts and their active compounds. The studies also provide scientific evidence for the reported ethnomedicinal purposes of these medicinal plants.

## References

[1] Worldometer Latin America and the Caribbean Population. https://www.worldometers.info/world-population/latin-america-and-the-caribbean-population/.

[2] Halpern B., Louzada M.L.D.C., Aschner P., Gerchman F., Brajkovich I., Faria-Neto J.R., Polanco F.E., Montero J., Juliá S.M.M., Lotufo P.A. (2021). Obesity and COVID-19 in Latin America: A tragedy of two pandemics-official document of the Latin American Federation of Obesity Societies. Obes. Rev..

[3] Macinko J., Andrade F.C.D., Nunes B.P., Guanais F.C. (2019). Primary care and multimorbidity in six Latin American and Caribbean countries. Rev. Panam. Salud Publica.

[4] Calazans J.A., Queiroz B.L. (2020). The adult mortality profile by cause of death in 10 Latin American countries (2000–2016). Rev. Panam. Salud Publica.

[5] Bilal U., Hessel P., Perez-Ferrer C., Michael Y.L., Alfaro T., Tenorio-Mucha J., Friche A.A.L., Pina M.F., Vives A., Quick H. (2021). Life expectancy and mortality in 363 cities of Latin America. Nat. Med..

[6] Chen Y., Freedman N.D., Rodriquez E.J., Shiels M.S., Napoles A.M., Withrow D.R., Spillane S., Sigel B., Perez-Stable E.J., Berrington de González A. (2020). Trends in premature deaths among adults in the United States and Latin America. JAMA Netw. Open.

[7] Mata R., Figueroa M., Navarrete A., Rivero-Cruz I. (2019). Chemistry and biology of selected Mexican medicinal plants. Prog. Chem. Org. Nat. Prod..

[8] Aguayo-Morales H., Sierra-Rivera C.A., Claudio-Rizo J.A., Cobos-Puc L.E. (2023). Horsetail (*Equisetum hyemale*) extract accelerates wound healing in diabetic rats by modulating IL-10 and MCP-1 release and collagen synthesis. Pharmaceuticals.

[9] Bustos-Gómez C.I., Gasca-Martínez D., Yáñez-Barrientos E., Hidalgo-Figueroa S., Gonzalez-Rivera M.L., Barragan-Galvez J.C., Zapata-Morales J.R., Isiordia-Espinoza M., Corrales-Escobosa A.R., Alonso-Castro A.J. (2022). Neuropharmacological activities of *Ceiba aesculifolia* (Kunth) Britten & Baker f (Malvaceae). Pharmaceuticals.

[10] Donadel G., Dalmagro M., de Oliveira J.A.B., Zardeto G., Pinc M.M., Hoscheid J., Alberton O., Belettini S.T., Jacomassi E., Gasparotto Junior A. (2022). Safety investigations of two formulations for vaginal use obtained from *Eugenia uniflora* L. leaves in female rats. Pharmaceuticals.

[11] Silva J.H.D., Abreu L.C.L., Ferrari R., Quintana C.Y.P., Barros E.G.O., Cordeiro N.M., Pontes B., Sousa V.P., Cabral L.M., Fernandes P.D. (2022). Copaiba oil resin exerts an additive effect to Babassu oil on behavioral changes in human endometriotic cell cultures. Pharmaceuticals.

[12] Ximenes T.V.N., Carvalho R., Bonfá I.S., Santos V.S., Candeloro L., Alves F.M., Silva D.B., Carollo C.A., Gielow K.C.F., Silva-Filho S.E. (2022). *Baccharis trimera* infusion reduces macrophages activation and high-fat diet-induced metabolic disorders in mice. Pharmaceuticals.

[13] Guilhon C.C., Minho A.S., Pouliot M., Boylan F., Fernandes P.D. (2022). *Tibouchina granulosa* leaves present anti-inflammatory effect. Pharmaceuticals.

[14] Campos-Xolalpa N., Esquivel-Campos A.L., Martínez-Casares R.M., Pérez-Gutiérrez S., Pérez-Ramos J., Sánchez-Mendoza E. (2022). Anti-Inflammatory activity of piquerol isolated from *Piqueria trinervia* Cav. Pharmaceuticals.

[15] Furman D., Campisi J., Verdin E., Carrera-Bastos P., Targ S., Franceschi C., Ferrucci L., Gilroy D.W., Fasano A., Miller G.W. (2019). Chronic inflammation in the etiology of disease across the life span. Nat. Med..

[16] Cravens A., Payne J., Smolke C.D. (2019). Synthetic biology strategies for microbial biosynthesis of plant natural products. Nat. Commun..

[17] Alonso-Castro A.J., Ruiz-Noa Y., Martínez-de la Cruz G.C., Ramírez-Morales M.A., Deveze-Álvarez M.A., Escutia-Gutiérrez R., Carranza-Álvarez C., Domínguez F., Maldonado-Miranda J.J., Ruiz-Padilla A.J. (2022). Factors and practices associated with self-medicating children among Mexican parents. Pharmaceuticals.

[18] Al-Imam A., Motyka M.A., Hoffmann B., Magowska A., Michalak M. (2023). Infoveillance and Critical Analysis of the Systematically Reviewed Literature on Dimethyltryptamine and the “God Molecule”. Pharmaceuticals.

[19] Salazar-Gómez A., Alonso-Castro A.J. (2022). Medicinal plants from Latin America with wound healing activity: Ethnomedicine, phytochemistry, preclinical and clinical studies—A review. Pharmaceuticals.

[20] Barreto Linhares L.P.M., Pereira B.V.N., Dantas M.K.G., Bezerra W.M.D.S., Viana-Marques D.A., de Lima L.R.A., Sette-de-Souza P.H. (2022). *Schinopsis brasiliensis* Engler-Phytochemical properties, biological activities, and ethnomedicinal use: A scoping review. Pharmaceuticals.

